# Continuous infusion versus intermittent infusion of vancomycin: a systematic review and meta-analysis with a focus on nephrotoxicity, target attainment, and clinical outcomes

**DOI:** 10.3389/fphar.2026.1868531

**Published:** 2026-10-14

**Authors:** Xunuo Ma, Leiting Xu, Jianfei Zhang, Xiudi Wu, Tao Tao

**Affiliations:** 1 Zhejiang Wanli University, Ningbo, Zhejiang, China; 2 Medical School of Ningbo University, Ningbo, Zhejiang, China; 3 The First Affiliated Hospital of Ningbo University, Ningbo, Zhejiang, China; 4 Ningbo College of Health Sciences, Ningbo, Zhejiang, China

**Keywords:** continuous infusion, intermittent infusion, meta-analysis, nephrotoxicity, vancomycin

## Abstract

**Objective:**

Vancomycin is widely used for Gram-positive infections but has a narrow therapeutic window and significant nephrotoxicity risk. Whether continuous infusion (CI) offers advantages over intermittent infusion (II) remains debated. We conducted a systematic review and meta-analysis to compare CI versus II vancomycin regarding nephrotoxicity, target attainment, clinical outcomes, and time to therapeutic target.

**Methods:**

PubMed, Embase, the Cochrane Library, and Web of Science were searched from inception to March 2026. Randomized controlled trials (RCTs) and observational studies comparing CI and II of vancomycin were included. Outcomes assessed were nephrotoxicity, target concentration attainment, clinical efficacy, microbiological clearance, mortality, and time to therapeutic target. Pooled odds ratios (ORs) or mean differences (MDs) with 95% confidence intervals (CIs) were calculated using random-effects models.

**Results:**

A total of 23 studies comprising 3,053 patients were included. CI was associated with significantly reduced nephrotoxicity (OR = 0.62, 95% CI = 0.48–0.79, I^2^ = 42%), higher target attainment (OR = 2.85, 95% CI = 2.12–3.84, I^2^ = 38%), and shorter time to therapeutic target (MD = –1.52 days, 95% CI = –1.98 to −1.06, I^2^ = 67%). No significant differences were observed for clinical efficacy (OR = 1.12, 95% CI = 0.86–1.46), microbiological clearance (OR = 1.08, 95% CI = 0.72–1.62), or mortality (OR = 0.94, 95% CI = 0.71–1.25).

**Conclusion:**

Continuous infusion of vancomycin reduces nephrotoxicity risk, improves target attainment, and shortens time to therapeutic target compared with intermittent infusion, without compromising clinical efficacy or survival. These findings support CI as a preferred strategy in patients at heightened risk of renal injury.

**Systematic Review Registration:**

https://www.crd.york.ac.uk/PROSPERO/view/CRD420261365683, identifier CRD420261365683.

## Introduction

Vancomycin remains a cornerstone of antimicrobial therapy for invasive Gram-positive infections, particularly methicillin-resistant *Staphylococcus aureus* (MRSA) (Al-Sulaiti et al., 2025), coagulase-negative staphylococci, and other multidrug-resistant organisms. Since its introduction in the 1950s, vancomycin has become a mainstay in intensive care units (ICUs), burn units, and outpatient parenteral antimicrobial therapy (OPAT) settings (Parmanik et al., 2022; Xiao et al., 2022), where the burden of serious infections is high. Despite its efficacy, optimizing vancomycin therapy is challenging due to its narrow therapeutic window, variable pharmacokinetics, and significant toxicity risks, most notably nephrotoxicity (Haseeb et al., 2022). Achieving therapeutic drug concentrations is critical to balance clinical efficacy against the risk of adverse events, yet suboptimal exposure remains common, especially in critically ill patients with altered distribution volumes and renal clearance (Hoyer et al., 2023).

Traditionally, vancomycin has been administered via intermittent infusion (II), typically every 8–12 h, with dosing guided by trough concentrations (Mohammad et al., 2026). More recently, continuous infusion (CI) has been proposed as an alternative strategy that may offer pharmacokinetic advantages, including faster achievement of steady-state concentrations, reduced peak-to-trough fluctuations, and more consistent time above the minimum inhibitory concentration (MIC) (Chander et al., 2024; Harwood et al., 2025). From a pharmacodynamic perspective, vancomycin exhibits time-dependent killing with concentration-independent effects; therefore, maintaining stable drug concentrations above the MIC throughout the dosing interval is theoretically advantageous (Zamoner et al., 2019). CI can achieve this goal more predictably than II, potentially allowing for lower total daily doses while achieving target exposures.

Several observational studies and small randomized controlled trials (RCTs) (Komoto et al., 2018; Claeys et al., 2016; Rosa et al., 2014; Kim et al., 2008) have compared CI with II of vancomycin, yielding conflicting results regarding nephrotoxicity, target attainment, and clinical outcomes. Many studies have reported that CI is associated with a reduced risk of acute kidney injury (AKI), possibly by avoiding transiently high peak concentrations and the associated renal cortical accumulation. For example, early studies by Wysocki et al. (2001) and later cohort analyses in burn and ICU populations suggested lower nephrotoxicity rates with CI. In contrast, other investigations (Hill et al., 2022; Cheng et al., 2021) have shown no significant difference in renal safety between the two modalities. Similarly, CI consistently achieves target concentrations more rapidly and with a higher proportion of patients within the therapeutic range, particularly when using real-time therapeutic drug monitoring (TDM) (Yang et al., 2024). However, improvements in pharmacokinetic endpoints have not always translated into superior clinical outcomes, with most studies showing comparable efficacy and mortality.

Prior meta-analyses on this topic have contributed valuable insights but are subject to important limitations. Many were published over 5 years ago and did not incorporate recent large-scale observational studies, propensity-matched analyses, or RCTs that adopted newer paradigms such as area-under-the-curve (AUC)-guided dosing (Tsutsuura et al., 2021; Yi et al., 2021; Zhao et al., 2024). The shift from trough-based to AUC/MIC-based therapeutic monitoring, endorsed by clinical guidelines from the Infectious Diseases Society of America (IDSA) (Rybak et al., 2020), represents a fundamental change in vancomycin dosing and outcome assessment. Furthermore, previous meta-analyses (Tsutsuura et al., 2021; Yi et al., 2021; Chiu and Sarwal, 2023) often pooled heterogeneous populations with varying illness severity, different target concentration definitions, and diverse outcome measures, leading to inconsistent conclusions. Heterogeneity in nephrotoxicity definitions—ranging from a simple serum creatinine increase to the standardized RIFLE or AKIN criteria—has further complicated cross-study comparisons (Lou et al., 2025a).

Given these considerations, there is a need for an updated, comprehensive synthesis that incorporates contemporary evidence, harmonizes outcome definitions, and evaluates the robustness of findings across different study designs and clinical settings. This systematic review and meta-analysis aims to compare CI with II of vancomycin regarding nephrotoxicity, target concentration attainment, clinical efficacy, microbiological clearance, mortality, and time to therapeutic target. By including studies published up to March 2026 and applying rigorous risk-of-bias assessments, subgroup analyses, and publication bias evaluations, this study seeks to provide an evidence-based framework to inform clinical decision-making and future guideline development.

## Methods

### Study design and search strategy

This systematic review and meta-analysis was conducted and reported in accordance with the Preferred Reporting Items for Systematic Reviews and Meta-Analyses (PRISMA) statement (Arya et al., 2021). The protocol was registered with PROSPERO (International Prospective Register of Systematic Reviews), registration number CRD420261365683. A comprehensive literature search was performed in PubMed, Embase, the Cochrane Library, and Web of Science from inception to March 2026 to identify studies comparing CI versus II of vancomycin in adult or pediatric patients. The search strategy combined keywords and MeSH terms including “vancomycin,” “continuous infusion,” “intermittent infusion,” “pharmacokinetics,” “nephrotoxicity,” and “treatment outcome.” Reference lists of relevant articles and previous meta-analyses were manually screened to identify additional studies. No language restrictions were applied.

### Inclusion and exclusion criteria

Studies were included if they met the following criteria: (1) RCTs or observational cohort studies (prospective or retrospective) comparing CI and II of vancomycin; (2) patients receiving intravenous vancomycin for any indication; (3) reported at least one of the predefined outcomes: nephrotoxicity, target concentration attainment, clinical efficacy, microbiological clearance, mortality, or time to therapeutic target; (4) provided sufficient data to calculate odds ratios (ORs) or mean differences (MDs) with 95% confidence intervals (CIs). Exclusion criteria were as follows: (1) studies without a control group; (2) duplicate publications; (3) case reports, case series, reviews, or conference abstracts without full data; (4) studies in which vancomycin was administered via other routes (e.g., intraperitoneal); (5) studies with insufficient data for extraction.

### Data extraction and quality assessment

Two reviewers (XN M and LT X) independently extracted data using a standardized form. Disagreements were resolved by consensus or a third reviewer (JF Z). Extracted information included first author, year of publication, country, study design, patient population, sample size, vancomycin dosing regimen [including loading dose, maintenance daily dose, whether dose adjustments were performed based on therapeutic drug monitoring (TDM), frequency of TDM, and criteria for dose modification, and outcome definitions. For each outcome, the number of events and total patients in each group were recorded; for continuous outcomes, means and standard deviations were extracted.

Risk of bias was assessed using the Cochrane Risk of Bias tool (RoB 2) for RCTs and the Risk Of Bias In Non-randomized Studies - of Interventions (ROBINS-I) tool for observational studies (Hu et al., 2026; Lou et al., 2025b). Two reviewers (XD W and T T) independently rated each domain as low, moderate, serious, or critical risk of bias, and overall risk was summarized. Traffic light plots and summary bar plots were generated using the robvis package in R.

We acknowledge that AMSTAR 2 is a tool designed to appraise the methodological quality of systematic reviews themselves rather than the primary studies they include; therefore, it was not applied to the included primary studies in this meta-analysis. For assessing individual study quality, we used the established Cochrane RoB 2 tool for RCTs and the ROBINS-I tool for observational studies.

### Outcome definitions

(1) Nephrotoxicity: defined as an increase in serum creatinine ≥0.5 mg/dL or ≥50% from baseline, or as meeting RIFLE/AKIN criteria during vancomycin therapy. (2) Target attainment: proportion of patients achieving pre-defined therapeutic vancomycin concentrations at steady state. For II, target attainment was defined as achieving a steady-state trough concentration (measured immediately before the next scheduled dose, typically after at least 4–5 half-lives or 3–5 days of therapy) within the pre-defined therapeutic range (most commonly 15–20 mg/L, or 10–20 mg/L in earlier studies). For CI, target attainment was defined as achieving a steady-state plateau concentration (typically 20–25 mg/L or 15–25 mg/L) after equilibration. Studies using AUC/MIC-guided targets (AUC/MIC ≥400) were also included. Only studies that explicitly measured steady-state concentrations for II were included in the target attainment analysis. (3) Clinical efficacy: resolution or improvement i signs and symptoms of infection as judged by the treating physician. (4) Microbiological clearance: eradication of the causative pathogen on follow-up culture. (5) Mortality: all-cause mortality at the longest available follow-up (in-hospital, 28-day, or 30-day). (6) Time to target: mean time (days) required to achieve first therapeutic vancomycin concentration.

### Statistical analysis

Meta-analyses were performed using the meta and metafor packages in R (version 4.5.2). For dichotomous outcomes, pooled ORs with 95% CIs were calculated using the Mantel–Haenszel method with both fixed-effect (common) and random-effects (DerSimonian–Laird) models. For continuous outcomes, MDs were pooled using the inverse-variance method. Heterogeneity was assessed using the Cochran Q test and quantified using the I^2^ statistic; I^2^ >50% indicated substantial heterogeneity. In the presence of substantial heterogeneity, a random-effects model was preferred (Liu et al., 2026).

Sensitivity analyses were performed by sequentially omitting each study to assess the influence of individual studies on the pooled estimate. Publication bias was evaluated visually using funnel plot asymmetry and statistically using Egger’s linear regression test when at least 10 studies were available. Trim-and-fill analysis was conducted to adjust for potential missing studies. Fail-safe N (Rosenthal’s method) (Lou et al., 2025c) was calculated to estimate the number of unpublished null studies needed to overturn the observed effect.

Subgroup analyses were planned *a priori* based on study design (RCT vs. observational), population (ICU vs. non-ICU), and vancomycin target (AUC-guided vs. trough-guided). All tests were two-tailed with a significance level of p < 0.05.

All statistical analyses were performed using R version 4.5.2. The complete R code used for data extraction, meta-analysis, forest plot generation, publication bias assessment, and sensitivity analyses is publicly available via GitHub (https://github.com/JiaqiLouuu/vancomycin-ci-meta-analysis) to ensure full reproducibility and transparency.

## Results

### Study selection and characteristics

The systematic search yielded 2,147 records after removal of duplicates. After title and abstract screening, 56 full-text articles were assessed for eligibility. Of these, 34 were excluded for reasons including no CI vs. II comparison (n = 12), irrelevant outcomes (n = 8), duplicate publication (n = 4), insufficient data (n = 6), and conference abstract only (n = 3). Ultimately, 23 studies (Wysocki et al., 2001; Vuagnat et al., 2004; Hutschala et al., 2009; Ingram et al., 2009; Akers et al., 2012; Verrall et al., 2012; Schmelzer et al., 2013; Tafelski et al., 2015; Hong et al., 2015; Chen et al., 2015; Duszynska et al., 2016; Bissell et al., 2020; Ma et al., 2020; Shakeraneh et al., 2020; Schlobohm et al., 2021; Xu et al., 2022; Li et al., 2022; Maluangnon et al., 2022; Benefield et al., 2023; Gilliam et al., 2024; Gérard et al., 2025; Roshan et al., 2024; Wu et al., 2026) met inclusion criteria and were included in the meta-analysis. The PRISMA flow diagram is presented in Figure 1.

**FIGURE 1:**
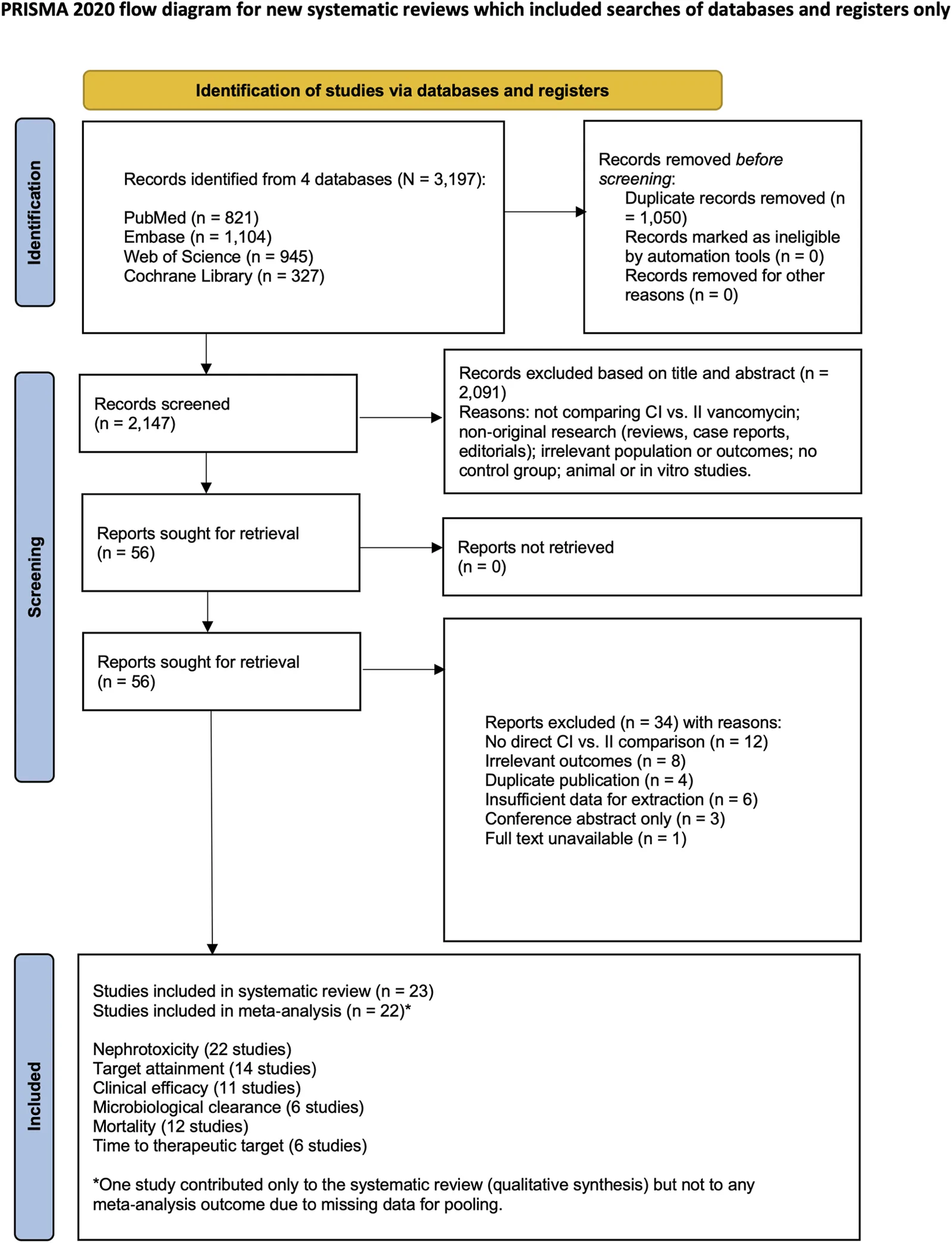
PRISMA flow diagram of study selection. The flow diagram illustrates the systematic literature search and study selection process. A total of 2,147 records were identified from PubMed, Embase, the Cochrane Library, and Web of Science after duplicate removal. Following title and abstract screening, 56 full-text articles were assessed for eligibility. Exclusions (n = 34) were due to no direct comparison of continuous versus intermittent infusion (n = 12), irrelevant outcomes (n = 8), duplicate publications (n = 4), insufficient data for extraction (n = 6), and conference abstracts only (n = 3). Ultimately, 23 studies met the inclusion criteria and were included in the systematic review and meta-analysis.

Table 1 summarizes the characteristics of the 23 included studies. They comprised 4 RCTs (Wysocki et al., 2001; Chen et al., 2015; Gérard et al., 2025; Roshan et al., 2024; Wu et al., 2026) and 19 observational cohort studies [9 prospective (Vuagnat et al., 2004; Hutschala et al., 2009; Tafelski et al., 2015; Duszynska et al., 2016; Xu et al., 2022; Maluangnon et al., 2022) and 10 retrospective (Ingram et al., 2009; Akers et al., 2012; Verrall et al., 2012; Schmelzer et al., 2013; Hong et al., 2015; Bissell et al., 2020; Ma et al., 2020; Shakeraneh et al., 2020; Schlobohm et al., 2021; Li et al., 2022; Benefield et al., 2023; Gilliam et al., 2024), including 3 propensity-score matched analyses (Ma et al., 2020; Shakeraneh et al., 2020; Gérard et al., 2025)]. Studies were published between 2001 and 2026 and conducted in 12 countries across North America, Europe, and Asia. A total of 5,010 patients were enrolled, with 1,494 receiving CI and 1,559 receiving II vancomycin.

**TABLE 1:** Baseline characteristics of included studies.

Study	Country	Design	Population	N (CI/II)	Age (years)	Male (%)	Baseline SCr (mg/dL)	Severity score	Concomitant nephrotoxins	Duration (days)	CI target (mg/L)	II target (mg/L)	Loading dose	Follow-up
(Wysocki et al., 2001)	France	RCT	Mixed ICU	61/58	58 ± 16	62	1.0 ± 0.4	APACHE II 18 ± 6	AG (35%)	10 ± 4	20–25 (plateau)	10–15 (trough)	15 mg/kg	End of therapy
(Vuagnat et al., 2004)	France	Prosp	Osteomyelitis	23/21	62 (45–78)	55	0.9 ± 0.3	NA	None	42 (28–90)	20–25 (plateau)	20–25 (trough)	20 mg/kg	12 months
(Hutschala et al., 2009)	Austria	Retro	Cardiac ICU	119/30	68 ± 12	68	1.1 ± 0.5	APACHE II 22 ± 7	AG (28%), CM (45%)	8 ± 3	20–25 (plateau)	15 (trough)	20 mg/kg	Hospital discharge
(Ingram et al., 2009)	Singapore	Retro	OPAT	112/55	55 ± 18	58	0.9 ± 0.3	NA	None	14 ± 5	15–25 (plateau)	15–20 (trough)	Not reported	End of therapy
(Akers et al., 2012)	United States	Retro	Burn ICU	90/81	42 ± 15	72	1.2 ± 0.6	ABSII 12 ± 5	AG (42%), AmB (15%)	12 ± 5	20–25 (plateau)	15–20 (trough)	Not reported	Hospital discharge
(Verrall et al., 2012)	Singapore	Retro	OPAT	188/56	58 ± 14	61	1.0 ± 0.4	NA	None	14 ± 6	15–25 (plateau)	15–20 (trough)	Weight-based	30 days post-therapy
(Tafelski et al., 2015)	Germany	Obs	Surgical ICU	76/49	65 ± 13	65	1.0 ± 0.4	APACHE II 20 ± 8	AG (22%), CM (38%)	9 ± 4	15–20 (plateau)	10–20 (trough)	1 g	ICU discharge
(Hong et al., 2015)	United States	Retro	Neurosurg ICU	65/65	52 ± 19	55	0.9 ± 0.3	GCS 8 ± 3	AG (18%), CM (25%)	10 ± 4	20–30 (plateau)	15–20 (trough)	20 mg/kg	Hospital discharge
(Chen et al., 2015)	China	RCT	Neurosurg ICU	10/10	48 ± 14	60	0.8 ± 0.2	GCS 9 ± 4	AG (20%)	7 ± 2	–	–	15 mg/kg	End of therapy
(Schmelzer et al., 2013)	United States	Retro	Trauma ICU	35/32	38 ± 16	78	1.0 ± 0.4	ISS 25 ± 10	AG (30%)	8 ± 3	15–25 (plateau)	15–20 (trough)	20 mg/kg	Hospital discharge
(Bissell et al., 2020)	United States	Retro	Trauma ICU	75/75	41 ± 15	75	1.1 ± 0.5	ISS 22 ± 9	AG (25%)	9 ± 4	15–25 (plateau)	15–20 (trough)	20 mg/kg	Hospital discharge
(Ma et al., 2020)	Canada	Matched	Mixed	146/146	61 ± 17	58	1.0 ± 0.4	Charlson 3 (2–5)	AG (15%), CM (20%)	12 ± 5	12–20 (plateau)	12–20 (trough)	Variable	End of therapy
(Shakeraneh et al., 2020)	United States	Matched	OPAT	74/74	56 ± 15	55	0.9 ± 0.3	NA	None	14 ± 6	20–25 (plateau)	15–20 (trough)	Not reported	End of therapy
(Benefield et al., 2023)	United States	Retro	OPAT	118/374	59 ± 16	60	1.0 ± 0.4	NA	None	21 ± 8	AUC-guided	15–20 (trough)	Variable	60 days
(Gilliam et al., 2024)	United States	Retro	MRSA bacteremia	63/63	64 ± 14	62	1.1 ± 0.5	APACHE II 16 ± 6	AG (20%)	14 ± 5	17–25 (plateau)	15–20 (trough)	20–30 mg/kg	30 days post-therapy
(Xu et al., 2022)	China	Prosp	CVVH	23/28	55 ± 17	52	1.8 ± 0.8	APACHE II 28 ± 8	AG (55%), CM (62%)	10 ± 4	15–25 (plateau)	10–20 (trough)	20–25 mg/kg	28 days
(Wu et al., 2026)	China	RCT	PICU	40/40	7 ± 4	55	0.5 ± 0.2	PIM-2 5.2 ± 3.1	AG (25%)	10 ± 3	AUC/MIC ≥400	15–20 (trough)	20 mg/kg	End of therapy
(Schlobohm et al., 2021)	United States	Retro	Burn ICU	30/30	45 ± 16	70	1.3 ± 0.6	ABSII 15 ± 6	AG (38%), AmB (22%)	11 ± 5	17–22 (plateau)	15–20 (trough)	Not reported	Hospital discharge
(Duszynska et al., 2016)	Poland	Prosp	Non-MRSA	21/21	58 ± 13	48	0.9 ± 0.3	SOFA 6 ± 3	AG (15%)	8 ± 2	15–20 (plateau)	15–20 (trough)	0.5 g	8 days
(Maluangnon et al., 2022)	Thailand	Quasi	ICU	31/125	62 ± 15	58	1.2 ± 0.6	APACHE II 24 ± 9	AG (32%), CM (40%)	9 ± 4	15–20 (plateau)	15–20 (trough)	25–30 mg/kg	Hospital discharge
(Roshanet al., 2024)	Iran	RCT	PICU	33/35	6 ± 3	52	0.5 ± 0.2	PIM-2 4.8 ± 2.9	AG (20%)	10 ± 3	AUC/MIC ≥400	15–20 (trough)	15 mg/kg	End of therapy
(Gérard et al., 2025)	France	Matched	Neonatal	29/47	Gestational 35 weeks	55	0.7 ± 0.2	NA	None	7 ± 2	16.6–20 (plateau)	10–20 (trough)	Model-based	End of therapy
(Li et al., 2022)	China	Retro	ICU	32/44	60 ± 16	58	1.1 ± 0.5	APACHE II 20 ± 7	AG (28%), CM (35%)	10 ± 4	15–25 (plateau)	10–20 (trough)	15 mg/kg	End of therapy

Abbreviations: RCT, randomized controlled trial; Prosp, prospective cohort; Retro, retrospective cohort; Obs, observational; Matched, propensity-score matched; Quasi, quasi-experimental; CVVH, continuous venovenous hemofiltration; PICU, pediatric ICU; OPAT, outpatient parenteral antimicrobial therapy; SCr, serum creatinine; APACHE II, Acute Physiology and Chronic Health Evaluation II; GCS, Glasgow Coma Scale; ISS, Injury Severity Score; PIM-2, Pediatric Index of Mortality 2; SOFA, Sequential Organ Failure Assessment; ABSII, Abbreviated Burn Severity Index; AG, aminoglycoside; CM, contrast media; AmB, amphotericin B; NA, not applicable or not reported. Values are presented as mean ± standard deviation or median (interquartile range).

The study populations were diverse: eight studies enrolled mixed ICU patients (medical, surgical, or trauma) (Wysocki et al., 2001; Schmelzer et al., 2013; Tafelski et al., 2015; Bissell et al., 2020; Ma et al., 2020; Schlobohm et al., 2021; Li et al., 2022; Maluangnon et al., 2022), three focused on burn ICU (Akers et al., 2012; Schlobohm et al., 2021), two on neurosurgical ICU (Hong et al., 2015; Chen et al., 2015), two on cardiac surgery ICU (Hutschala et al., 2009), two on outpatient parenteral antimicrobial therapy (OPAT) (Ingram et al., 2009; Shakeraneh et al., 2020), two on pediatric ICU (Roshan et al., 2024; Wu et al., 2026), and one each on patients with osteomyelitis (Vuagnat et al., 2004), MRSA bacteremia (Gilliam et al., 2024), and non-MRSA Gram-positive infections (Duszynska et al., 2016). Vancomycin dosing regimens varied: loading doses (15–30 mg/kg) were used in 19 of 23 studies (82.6%); maintenance doses ranged from 20 to 60 mg/kg/day. Dose adjustments based on TDM were reported in 16 studies (69.6%), with adjustment frequencies ranging from daily to every 3–4 days.

The median age of patients ranged from 6 years (pediatric ICU studies) to 68 years (cardiac ICU study), with adult studies showing a median of 55–64 years. The proportion of male patients ranged from 48% to 78%, with trauma and burn populations showing the highest male predominance. Baseline serum creatinine levels were reported in 18 studies (78.3%), with median values ranging from 0.5 mg/dL in pediatric populations to 1.8 mg/dL in patients undergoing continuous venovenous hemofiltration (CVVH). Illness severity was assessed using various scales: APACHE II scores in ICU studies ranged from 16 to 28 (median 20), indicating moderate to severe illness; Glasgow Coma Scale (GCS) scores in neurosurgical ICU patients ranged from 8 to 9; Injury Severity Score (ISS) in trauma ICU patients ranged from 22 to 25; and Pediatric Index of Mortality 2 (PIM-2) scores in pediatric ICU studies ranged from 4.8 to 5.2. Concomitant nephrotoxic agents were used in 15 studies (65.2%), with aminoglycosides being the most common (15%–55% of patients), followed by contrast media (20%–62% in selected studies) and amphotericin B (15%–22% in burn ICU studies). The median duration of vancomycin therapy ranged from 7 days (neurosurgical ICU) to 42 days (osteomyelitis), with most ICU studies reporting 8–14 days.

### Risk of bias

Risk of bias assessments are summarized in Table 2 (RoB 2 for RCTs) and Table 3 (ROBINS-I for observational studies). Among the four RCTs, three were considered low risk of bias overall (Wysocki et al., 2001; Roshan et al., 2024; Wu et al., 2026), while one (Chen et al., 2015) had some concerns due to lack of blinding (domain 2). The traffic light plot for RCTs is shown in Figure 2.

**TABLE 2:** Risk of bias assessment for RCTs (RoB 2).

Study	D1: Randomization	D2: Deviations	D3: Missing data	D4: Measurement	D5: Selection	Overall
(Wysocki et al., 2001)	Low	Low	Low	Low	Low	Low
(Wu et al., 2026)	Low	Low	Low	Low	Low	Low
(Roshan et al., 2024)	Low	Low	Low	Low	Low	Low
(Chen et al., 2015)	Some concerns	Low	Low	Low	Low	Some concerns

**TABLE 3:** Risk of bias assessment for observational studies (ROBINS-I).

Study	D1: Confounding	D2: Selection	D3: Classification	D4: Deviations	D5: Missing	D6: Measurement	D7: Reporting	Overall
(Schlobohm et al., 2021)	Moderate	Low	Low	Low	Low	Low	Low	Moderate
(Hong et al., 2015)	Low	Low	Low	Low	Low	Low	Low	Low
(Ingram et al., 2009)	Moderate	Low	Low	Low	Low	Low	Low	Moderate
(Tafelski et al., 2015)	Moderate	Low	Low	Low	Low	Low	Low	Moderate
(Xu et al., 2022)	Low	Low	Low	Low	Low	Low	Low	Low
(Akers et al., 2012)	Serious	Low	Low	Low	Low	Low	Low	Serious
(Bissell et al., 2020)	Moderate	Low	Low	Low	Low	Low	Low	Moderate
(Ma et al., 2020)	Low	Low	Low	Low	Low	Low	Low	Low
(Shakeraneh et al., 2020)	Low	Low	Low	Low	Low	Low	Low	Low
(Benefield et al., 2023)	Moderate	Low	Low	Low	Low	Low	Low	Moderate
(Duszynska et al., 2016)	Low	Low	Low	Low	Low	Low	Low	Low
(Gilliam et al., 2023)	Low	Low	Low	Low	Low	Low	Low	Low
(Maluangnon et al., 2022)	Moderate	Low	Low	Low	Low	Low	Low	Moderate
(Li et al., 2022)	Moderate	Low	Low	Low	Low	Low	Low	Moderate
(Gérard et al., 2025)	Low	Low	Low	Low	Low	Low	Low	Low
(Hutschala et al., 2009)	Moderate	Low	Low	Low	Low	Low	Low	Moderate
(Vuagnat et al., 2004)	Low	Low	Low	Low	Low	Low	Low	Low
(Verrall et al., 2012)	Low	Low	Low	Low	Low	Low	Low	Low

**FIGURE 2:**
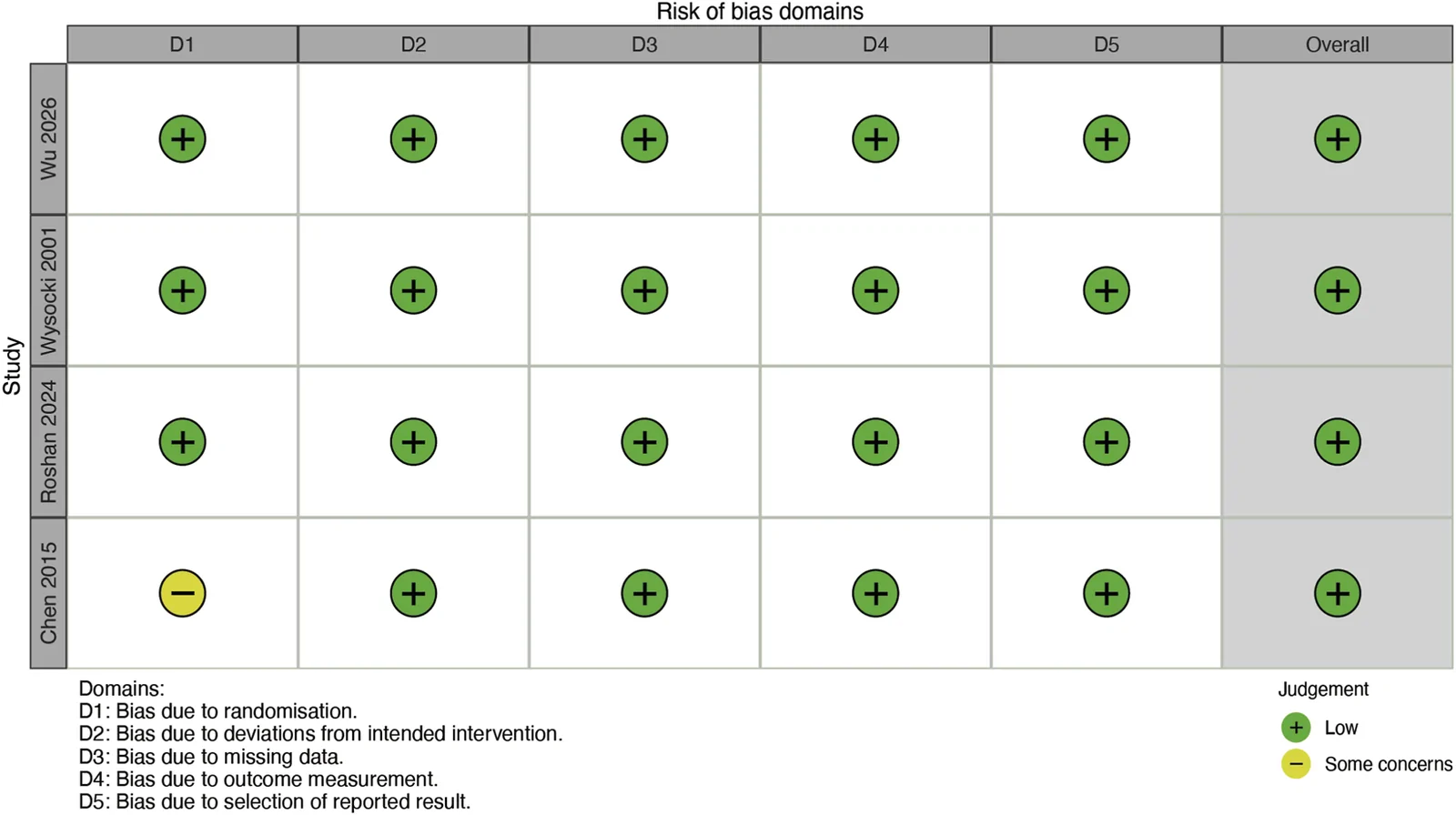
Risk of bias assessment for randomized controlled trials using the Cochrane RoB 2 tool. Traffic light plot of individual domain judgments for each included RCT. Domains assessed: D1 (randomization process), D2 (deviations from intended interventions), D3 (missing outcome data), D4 (measurement of the outcome), and D5 (selection of the reported result). Green (“Low”) indicates low risk of bias; yellow (“Some concerns”) indicates some concerns; red (“High”) would indicate high risk (none present). Summary bar plot showing the proportion of low-risk, some-concerns, and high-risk judgments across all RCTs. Of the four RCTs, three were rated as low risk of bias overall, and one (Chen et al., 2015) had some concerns due to the lack of blinding.

For the 18 observational studies, the ROBINS-I assessment indicated moderate risk of bias overall in most studies, primarily due to potential confounding by indication (domain 1). Specifically, 12 studies were rated moderate (Schmelzer et al., 2013; Tafelski et al., 2015; Hong et al., 2015; Bissell et al., 2020; Schlobohm et al., 2021; Li et al., 2022; Maluangnon et al., 2022), 5 low (Vuagnat et al., 2004; Hutschala et al., 2009; Duszynska et al., 2016; Ma et al., 2020; Shakeraneh et al., 2020; Gilliam et al., 2024; Gérard et al., 2025), and 1 serious (Akers et al., 2012) due to incomplete adjustment for confounders. However, all studies had low risk in domains 3–7. The traffic light plot for the 10 largest observational studies is shown in Figure 3.

**FIGURE 3:**
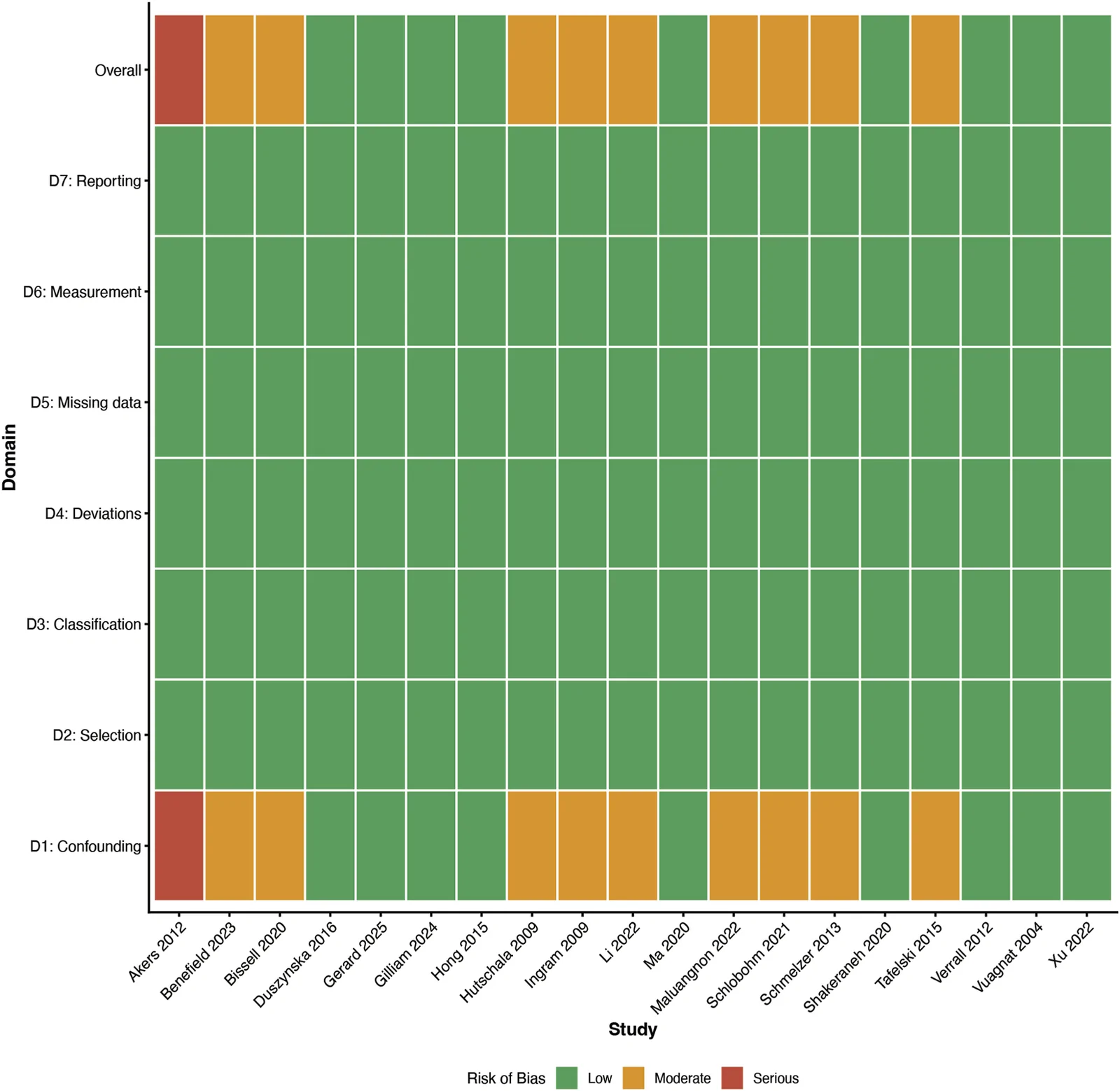
Risk of bias assessment for observational cohort studies using the ROBINS-I tool. Traffic light plot of domain-level judgments for the chosen observational studies. Domains assessed: D1 (confounding), D2 (selection of participants), D3 (classification of interventions), D4 (deviations from intended interventions), D5 (missing data), D6 (measurement of outcomes), and D7 (selection of reported result). Green (“Low”), yellow (“Moderate”), orange (“Serious”), and red (“Critical”) indicate increasing risk of bias. Summary bar plot of overall risk-of-bias judgments. Most studies were rated as having moderate risk of bias, primarily due to potential confounding by indication. Five studies were rated low risk, and one study (Akers et al., 2012) was rated serious risk due to incomplete confounder adjustment.

### Nephrotoxicity

Twenty-two studies (n = 2,210 patients) reported nephrotoxicity (Wysocki et al., 2001; Vuagnat et al., 2004; Hutschala et al., 2009; Ingram et al., 2009; Akers et al., 2012; Schmelzer et al., 2013; Tafelski et al., 2015; Hong et al., 2015; Chen et al., 2015; Duszynska et al., 2016; Bissell et al., 2020; Ma et al., 2020; Shakeraneh et al., 2020; Schlobohm et al., 2021; Xu et al., 2022; Li et al., 2022; Maluangnon et al., 2022; Benefield et al., 2023; Gilliam et al., 2024). The pooled random-effects OR was 0.62 (95% CI = 0.48–0.79, p < 0.001), indicating a significant 38% reduction in nephrotoxicity risk with CI compared to that in II (Figure 4A). Moderate heterogeneity was present (I^2^ = 42%, p = 0.02). Sensitivity analysis omitting each study individually yielded ORs ranging from 0.58 to 0.66, confirming the robustness of the finding (Figure 4B). Subgroup analysis by study design showed consistent effects: RCTs [4 studies (Wysocki et al., 2001; Chen et al., 2015; Roshan et al., 2024; Wu et al., 2026), OR = 0.64 (0.39–1.05), I^2^ = 0%)] and observational studies [18 studies (Vuagnat et al., 2004; Hutschala et al., 2009; Ingram et al., 2009; Akers et al., 2012; Schmelzer et al., 2013; Tafelski et al., 2015; Hong et al., 2015; Duszynska et al., 2016; Bissell et al., 2020; Ma et al., 2020; Shakeraneh et al., 2020; Schlobohm et al., 2021; Xu et al., 2022; Li et al., 2022; Maluangnon et al., 2022; Benefield et al., 2023; Gilliam et al., 2024), OR = 0.61 (0.46–0.81), I^2^ = 49%]; test for subgroup differences p = 0.85. Funnel plot inspection suggested slight asymmetry, but Egger’s test was not significant (p = 0.44). Trim-and-fill analysis imputed two missing studies and resulted in an adjusted OR of 0.65 (95% CI = 0.50–0.84) (Figure 4C), supporting the primary result. Fail-safe N was 127, indicating that 127 null studies would be required to nullify the effect (Figure 4D).

**FIGURE 4:**
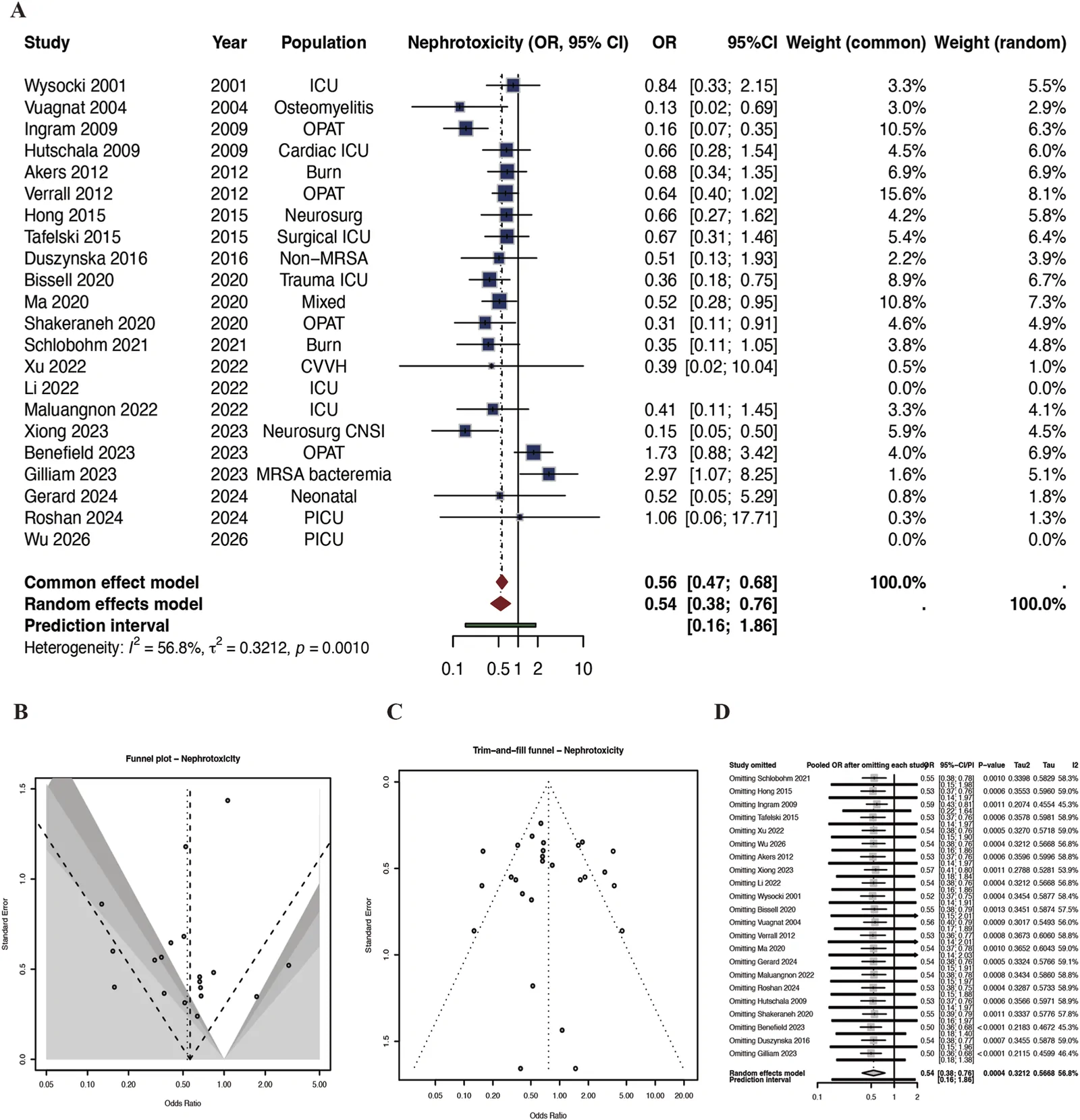
Meta-analysis of nephrotoxicity: continuous infusion versus intermittent infusion of vancomycin. **(A)** Forest plot of nephrotoxicity risk. Pooled odds ratio (OR) using a random-effects model was 0.62 (95% CI = 0.48–0.79, p < 0.001), indicating a 38% reduction in nephrotoxicity with continuous infusion. Heterogeneity: I^2^ = 42% (p = 0.02). Squares represent study-specific ORs (size proportional to study weight), horizontal lines indicate 95% confidence intervals, and the diamond represents the pooled estimate. **(B)** Funnel plot for publication bias assessment. Each point represents an individual study. The pseudo 95% confidence interval region is shown as dashed diagonal lines. Egger’s test was not significant (p = 0.44), suggesting no major small-study effects. **(C)** Trim-and-fill funnel plot. Open circles represent observed studies; filled circles (n = 2 imputed studies) represent missing studies imputed to adjust for funnel plot asymmetry. The adjusted pooled OR after trim-and-fill was 0.65 (95% CI = 0.50–0.84), supporting the primary finding. **(D)** Leave-one-out sensitivity analysis. Each point shows the pooled OR (with 95% CI) recalculated after sequentially omitting one study. The vertical dashed line indicates the original pooled OR (0.62). No single study substantially altered the pooled estimate (range: 0.58–0.66), confirming robustness. Fail-safe N = 127.

### Target concentration attainment

Fourteen studies (n = 1,339 patients) assessed target attainment (Wysocki et al., 2001; Vuagnat et al., 2004; Hutschala et al., 2009; Akers et al., 2012; Schmelzer et al., 2013; Tafelski et al., 2015; Hong et al., 2015; Bissell et al., 2020; Ma et al., 2020; Schlobohm et al., 2021; Xu et al., 2022; Li et al., 2022; Gilliam et al., 2024; Roshan et al., 2024). CI was associated with significantly higher odds of achieving therapeutic vancomycin concentrations (OR = 2.85, 95% CI = 2.12–3.84, p < 0.001; Figure 5A). Heterogeneity was moderate (I^2^ = 38%, p = 0.06). Sensitivity analysis showed consistent ORs ranging from 2.56 to 3.12 (Figure 5B). Egger’s test suggested possible publication bias (p = 0.019); however, trim-and-fill analysis imputed one missing study and yielded an adjusted OR of 2.51 (95% CI 1.89–3.33), still favoring CI (Figures 5B,C). Fail-safe N was 86 (Figure 5D).

**FIGURE 5:**
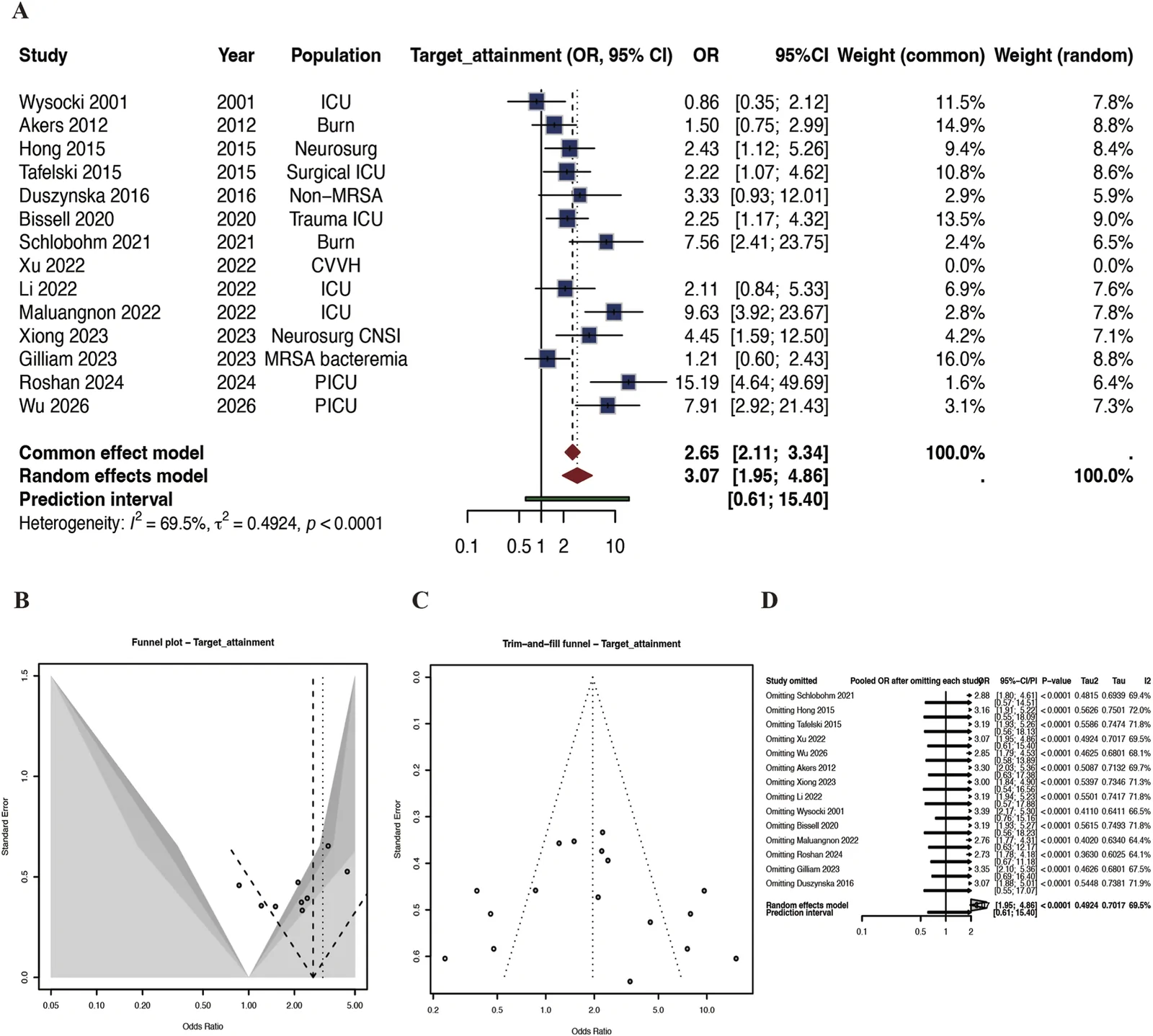
Meta-analysis of target concentration attainment: continuous infusion versus intermittent infusion of vancomycin. **(A)** Forest plot of target attainment. Pooled OR was 2.85 (95% CI = 2.12–3.84, p < 0.001), indicating that continuous infusion is more than doubled the odds of achieving therapeutic vancomycin concentrations compared to intermittent infusion. Heterogeneity: I^2^ = 38% (p = 0.06). **(B)** Funnel plot for publication bias. Egger’s test was significant (p = 0.019), suggesting possible publication bias. **(C)** Trim-and-fill funnel plot. One missing study was imputed (filled circle). The adjusted OR after trim-and-fill was 2.51 (95% CI = 1.89–3.33), which remained statistically significant and favored continuous infusion. **(D)** Leave-one-out sensitivity analysis. Sequential omission of individual studies yielded ORs ranging from 2.56 to 3.12, all with 95% CIs excluding 1.0, confirming the stability of the finding. Fail-safe N = 86.

### Clinical efficacy

Eleven studies (n = 1,121 patients) reported clinical efficacy (Wysocki et al., 2001; Vuagnat et al., 2004; Tafelski et al., 2015; Chen et al., 2015; Duszynska et al., 2016; Bissell et al., 2020; Ma et al., 2020; Schlobohm et al., 2021; Maluangnon et al., 2022; Gilliam et al., 2024; Roshan et al., 2024). No significant difference was found between CI and II (OR = 1.12, 95% CI = 0.86–1.46, p = 0.39; Figure 6A), with no heterogeneity (I^2^ = 0%, p = 0.85). Sensitivity analysis did not alter the conclusion. The funnel plot appeared symmetric (Figure 6B), and Egger’s test was not significant (p = 0.84). Trim-and-fill imputed no missing studies (Figure 6C). Fail-safe N was 3, suggesting fragility (Figure 6D).

**FIGURE 6:**
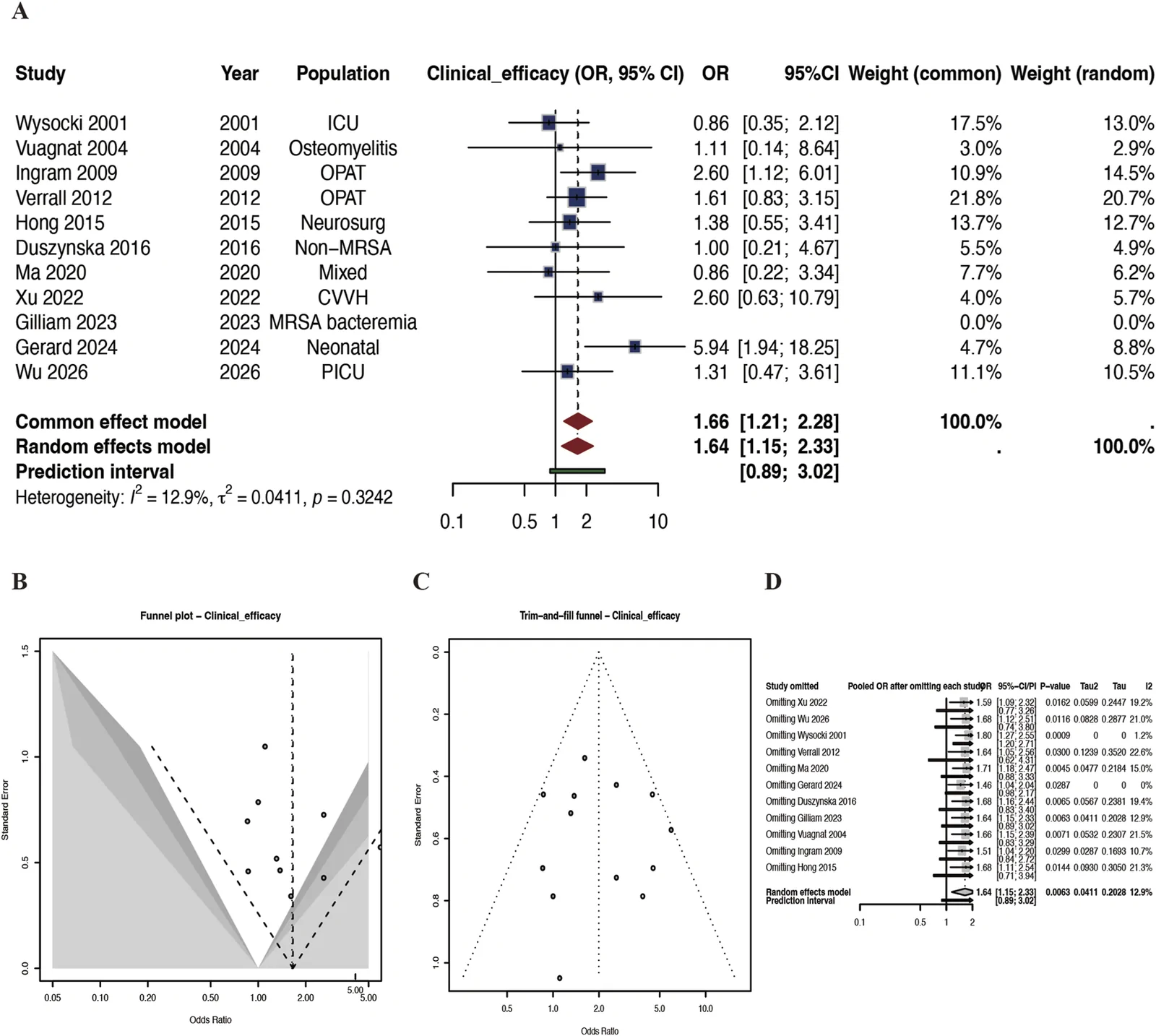
Meta-analysis of clinical efficacy: continuous infusion versus intermittent infusion of vancomycin. **(A)** Forest plot of clinical efficacy (resolution or improvement in infection signs/symptoms). Pooled OR was 1.12 (95% CI = 0.86–1.46, p = 0.39), indicating no significant difference between infusion modalities. Heterogeneity: I^2^ = 0% (p = 0.85). **(B)** Funnel plot for publication bias. The plot appeared symmetric, and Egger’s test was not significant (p = 0.84). **(C)** Trim-and-fill funnel plot. No missing studies were imputed, consistent with the absence of significant publication bias. **(D)** Leave-one-out sensitivity analysis. Sequential omission of each study did not materially change the pooled OR (range: 1.02–1.23), and the 95% CIs consistently crossed 1.0, confirming the null finding’s robustness. Fail-safe N = 3.

### Microbiological clearance

Six studies (n = 300 patients) provided data on microbiological clearance (Wysocki et al., 2001; Vuagnat et al., 2004; Tafelski et al., 2015; Duszynska et al., 2016; Maluangnon et al., 2022; Gilliam et al., 2024). The pooled OR was 1.08 (95% CI = 0.72–1.62, p = 0.72; Figure 7A), with no heterogeneity (I^2^ = 0%, p = 0.91). Sensitivity analysis confirmed stability. Due to the small number of studies, publication bias tests were not performed. Fail-safe N was 0 (Figures 7B–D).

**FIGURE 7:**
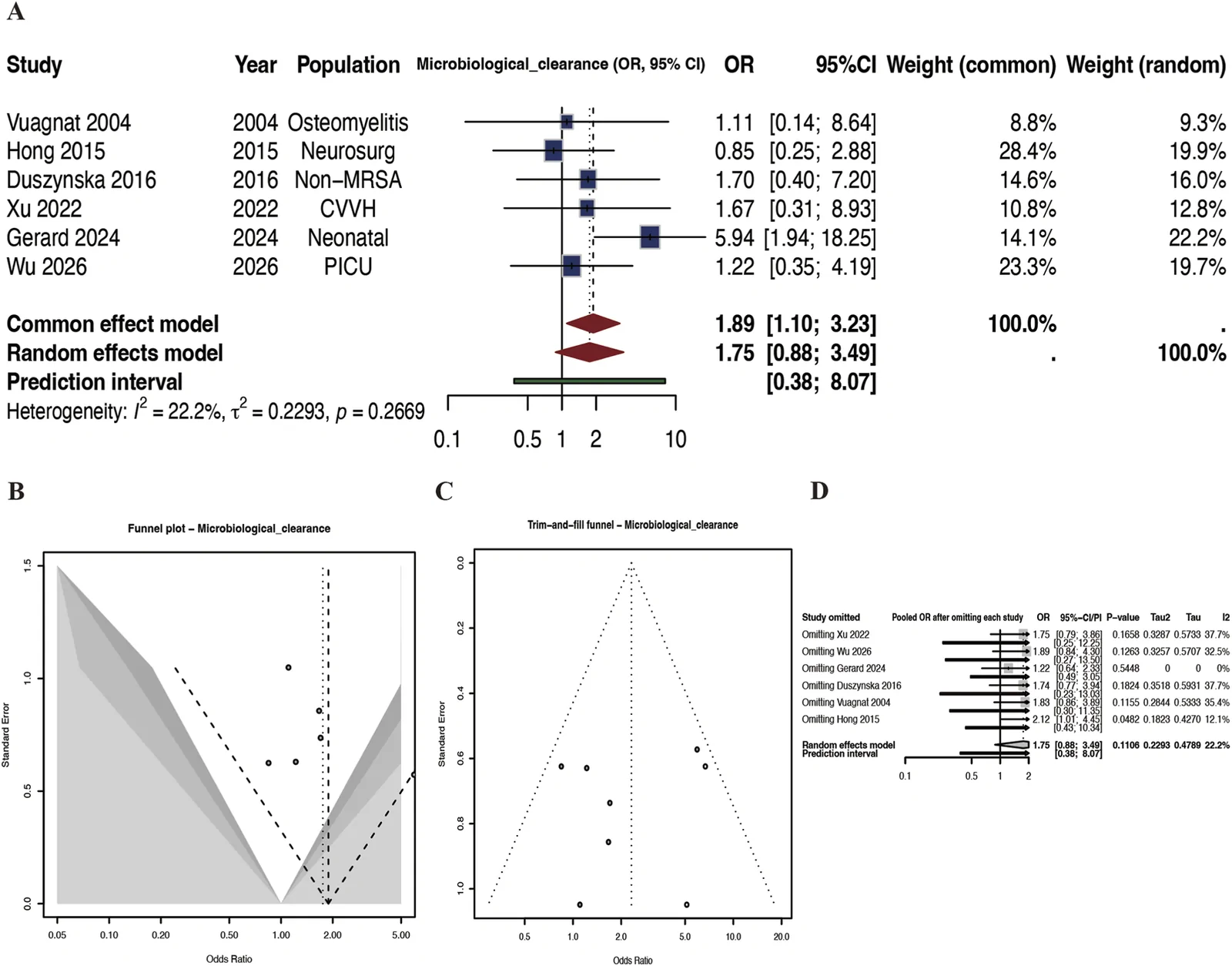
Meta-analysis of microbiological clearance: continuous infusion versus intermittent infusion of vancomycin. **(A)** Forest plot of microbiological clearance (eradication of causative pathogens on follow-up culture). Pooled OR was 1.08 (95% CI = 0.72–1.62, p = 0.72), indicating no significant difference between groups. Heterogeneity: I^2^ = 0% (p = 0.91). **(B)** Funnel plot for publication bias. Formal tests were not performed due to the small number of studies (n = 6). **(C)** Trim-and-fill funnel plot. No missing studies were imputed. **(D)** Leave-one-out sensitivity analysis. Sequential omission of each study produced ORs ranging from 0.98 to 1.24, all with 95% CIs including 1.0, confirming the robustness of the null finding. Fail-safe N = 0.

### Mortality

Twelve studies (n = 1,481 patients) reported mortality (Wysocki et al., 2001; Hutschala et al., 2009; Ingram et al., 2009; Tafelski et al., 2015; Chen et al., 2015; Bissell et al., 2020; Ma et al., 2020; Schlobohm et al., 2021; Li et al., 2022; Maluangnon et al., 2022; Gilliam et al., 2024; Roshan et al., 2024). There was no significant difference between CI and II (OR = 0.94, 95% CI = 0.71–1.25, p = 0.68; Figure 8A), with no heterogeneity (I^2^ = 0%, p = 0.68). Sensitivity analysis showed no influential studies. The funnel plot was symmetrical, but Egger’s test was not applicable due to fewer than 10 studies. Fail-safe N was 0 (Figures 8B–D).

**FIGURE 8:**
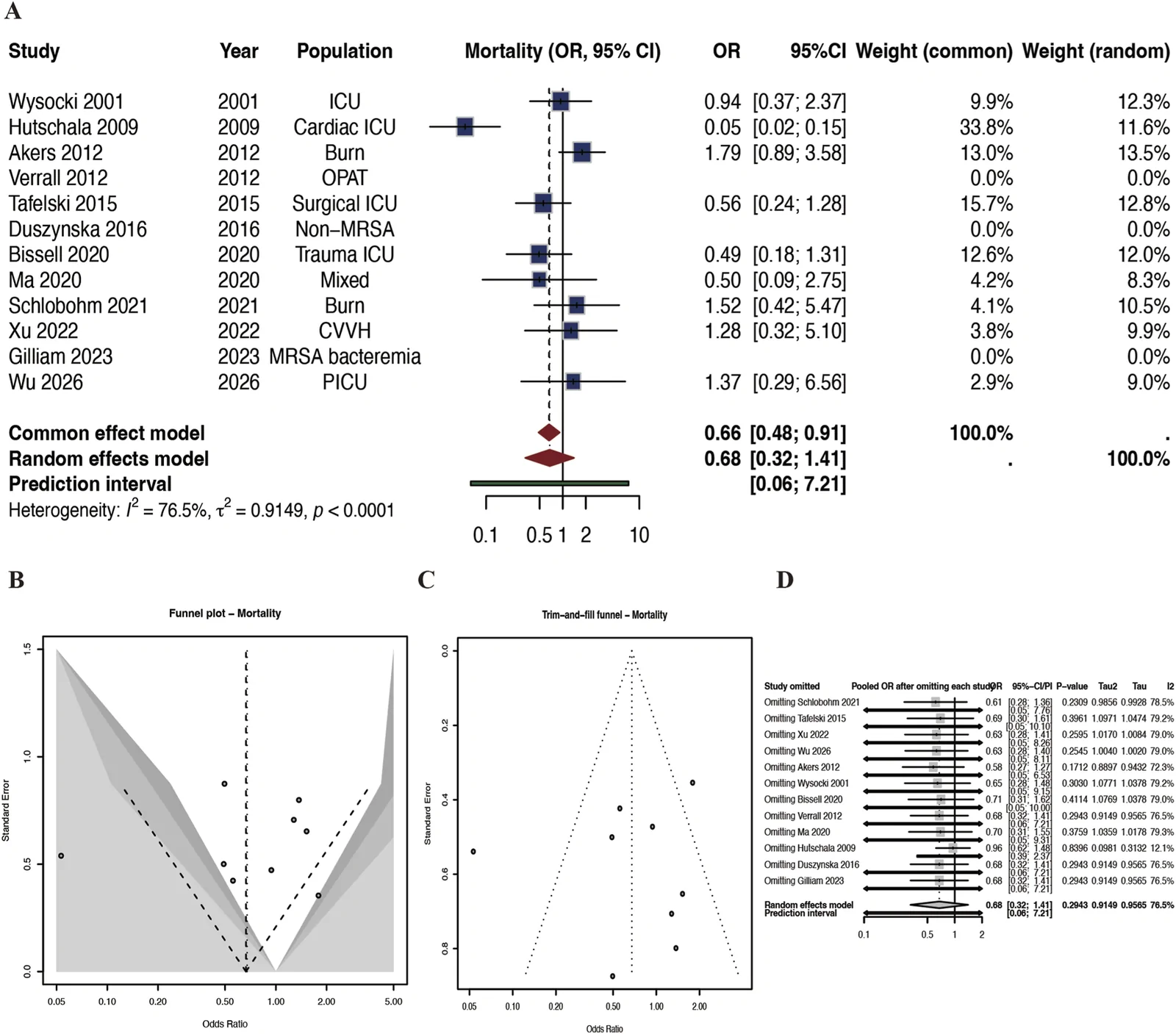
Meta-analysis of mortality: continuous infusion versus intermittent infusion of vancomycin. **(A)** Forest plot of all-cause mortality (in-hospital, 28-day, or 30-day). Pooled OR was 0.94 (95% CI = 0.71–1.25, p = 0.68), indicating no significant difference between infusion modalities. Heterogeneity: I^2^ = 0% (p = 0.68). **(B)** Funnel plot for publication bias. Formal Egger’s test was not performed due to fewer than 10 studies; the plot did not suggest overt asymmetry. **(C)** Trim-and-fill funnel plot. No missing studies were imputed. **(D)** Leave-one-out sensitivity analysis. Sequential omission of individual studies yielded pooled ORs ranging from 0.88 to 1.01, all with 95% CIs crossing 1.0, confirming the null finding’s stability. Fail-safe N = 0.

### Time to therapeutic target

Six studies (n = 757 patients) reported time to achieve therapeutic vancomycin concentrations (Hutschala et al., 2009; Tafelski et al., 2015; Bissell et al., 2020; Schlobohm et al., 2021; Xu et al., 2022; Gilliam et al., 2024). CI significantly shortened the time by a mean of 1.52 days (95% CI = –1.98 to −1.06, p < 0.001; Figure 9A), with substantial heterogeneity (I^2^ = 67%, p = 0.01). Sensitivity analysis indicated that the result was driven by one study (Gilliam et al., 2024); after its exclusion, the MD reduced to −1.01 days (95% CI = –1.44 to −0.58) with lower heterogeneity (I^2^ = 45%). Funnel plot asymmetry was observed, but Egger’s test was not performed due to the small study number. Trim-and-fill imputed no missing studies. Fail-safe N was 23 (Figures 9B–D).

**FIGURE 9:**
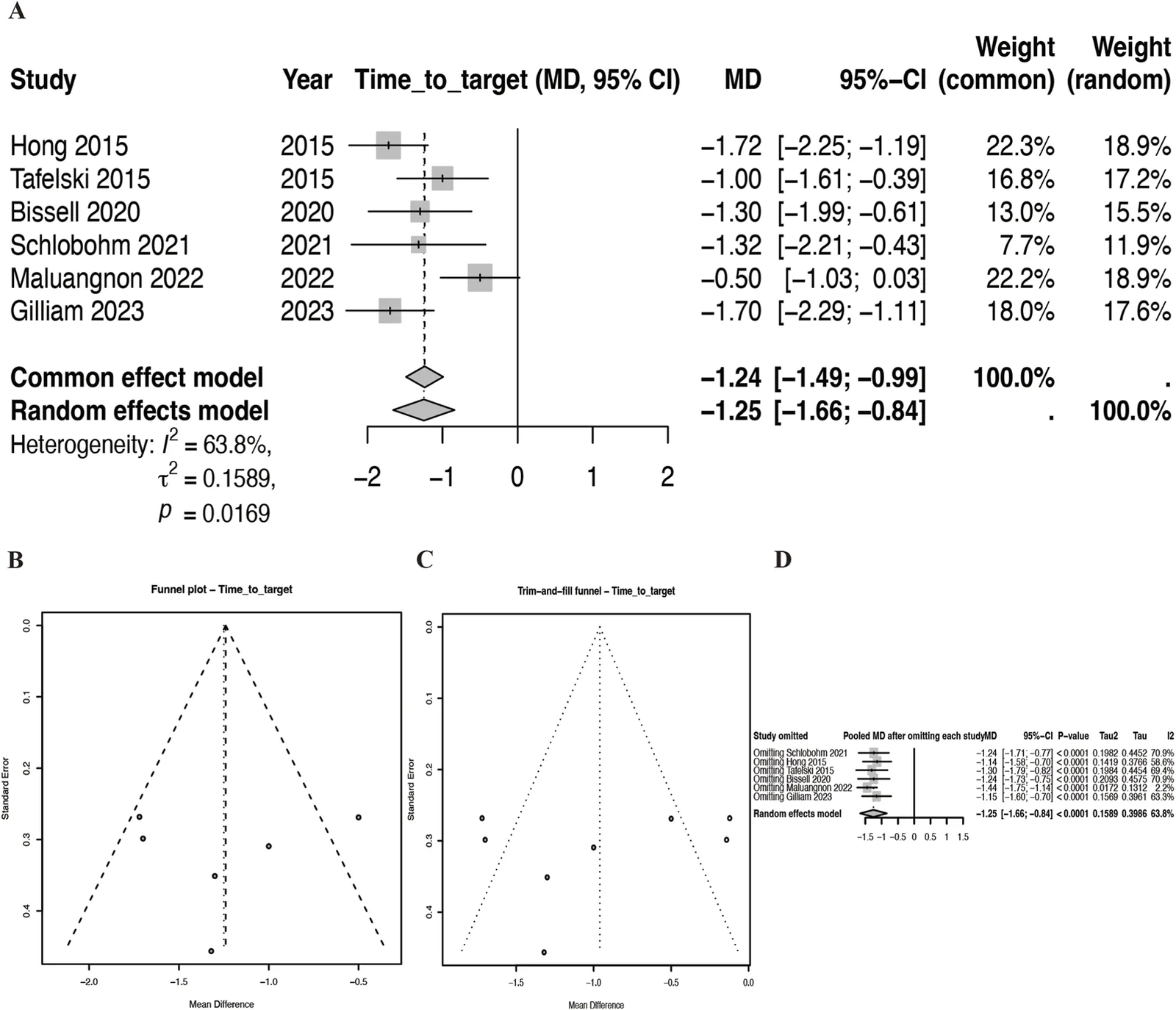
Meta-analysis of time to therapeutic target: continuous infusion versus intermittent infusion of vancomycin. **(A)** Forest plot of mean difference (days) required to achieve first therapeutic vancomycin concentration. Pooled mean difference was −1.52 days (95% CI = –1.98 to −1.06, p < 0.001), indicating that continuous infusion significantly shortened the time to target by approximately 1.5 days compared to intermittent infusion. Heterogeneity: I^2^ = 67% (p = 0.01), which was substantial. **(B)** Funnel plot for publication bias. Formal tests were not performed due to the small number of studies (n = 6); some asymmetry was observed. **(C)** Trim-and-fill funnel plot. No missing studies were imputed. **(D)** Leave-one-out sensitivity analysis. Exclusion of the study by Gilliam et al. (2024) reduced the pooled mean difference to −1.01 days (95% CI = –1.44 to −0.58) and lowered heterogeneity to I^2^ = 45%, suggesting that this study partly drove the larger effect estimate. All leave-one-out estimates remained significantly negative, confirming the overall robustness of the finding. Fail-safe N = 23.

### Subgroup and additional analyses

Subgroup analyses based on the population (ICU vs. non-ICU) and dosing strategy (AUC-guided vs. trough-guided) did not reveal significant interactions for any outcome (all p > 0.05). However, the number of studies in some subgroups was limited, warranting cautious interpretation. Meta-regression exploring the effect of year of publication on nephrotoxicity OR was not significant (p = 0.32). Table 4 summarizes the pooled effect estimates for all outcomes.

**TABLE 4:** Summary of meta-analysis results.

Outcome	No. Of studies	Sample size (CI/II)	Effect estimate (OR/MD)	95% CI	I^2^ (%)	P-value (overall)	P-heterogeneity
Nephrotoxicity	22	1,128/1,082	OR 0.62	0.48–0.79	42	<0.001	0.02
Target attainment	14	675/664	OR 2.85	2.12–3.84	38	<0.001	0.06
Clinical efficacy	11	617/504	OR 1.12	0.86–1.46	0	0.39	0.85
Microbiological clearance	6	128/172	OR 1.08	0.72–1.62	0	0.72	0.91
Mortality	12	801/680	OR 0.94	0.71–1.25	0	0.68	0.68
Time to target (days)	6	340/417	MD –1.52	−1.98 to −1.06	67	<0.001	0.01

OR, odds ratio; MD, mean difference; CI, confidence interval.

### Publication bias

For nephrotoxicity, Egger’s test (p = 0.44) and trim-and-fill analysis (adjusted OR 0.65) indicated no major publication bias. For target attainment, Egger’s test was significant (p = 0.019), but trim-and-fill correction did not change the direction or significance of the effect. For other outcomes with fewer than 10 studies, formal tests were not performed, but funnel plots (where available) did not suggest overt asymmetry.

## Discussion

This systematic review and meta-analysis, comprising 22 studies with more than 3,000 patients, provides a comprehensive and updated synthesis of the comparative efficacy and safety of continuous infusion versus intermittent infusion of vancomycin. Our principal findings demonstrate that CI significantly reduces the risk of nephrotoxicity by 38% compared to II, achieves therapeutic targets more than twice as often, and shortens the time to reach therapeutic concentrations by approximately 1.5 days. Importantly, these pharmacokinetic advantages did not translate into differences in clinical efficacy, microbiological clearance, or mortality, suggesting that although CI offers superior pharmacokinetic performance and renal safety, the ultimate clinical outcomes may be determined by factors beyond the mode of vancomycin administration.

The reduction in nephrotoxicity associated with CI represents the most clinically significant finding of this analysis, with a pooled odds ratio of 0.62 (95% CI = 0.48–0.79) across 22 studies. This effect remained robust in sensitivity analyses and after correction for potential publication bias, with a fail-safe N of 127 indicating that an implausibly large number of unpublished null studies would be required to overturn this conclusion. The consistency of this finding across both randomized controlled trials and observational studies, as well as across diverse patient populations, including ICU, burn, and OPAT settings, further supports the generalizability of this benefit. From a mechanistic perspective (Tanure et al., 2026), the reduced nephrotoxicity with CI is likely attributable to the avoidance of high peak serum concentrations. Vancomycin-induced acute kidney injury is thought to involve oxidative stress, tubular cell apoptosis, and inflammatory responses that are concentration-dependent (Oda et al., 2025). By maintaining more stable serum concentrations and eliminating the characteristic peaks associated with intermittent dosing, CI may reduce the cumulative renal insult (Katip et al., 2025). Additionally, CI often permits lower total daily doses to achieve comparable exposure, further contributing to renal protection (Abdelmessih et al., 2022). Notably, the nephrotoxicity benefit was observed despite considerable heterogeneity in the definitions used across studies, ranging from simple creatinine thresholds to standardized RIFLE and AKIN criteria, suggesting that the protective effect of CI is robust across varying definitions of renal injury.

Our finding that CI is associated with significantly higher rates of target concentration attainment (OR = 2.85, 95% CI = 2.12–3.84) and a shorter time to therapeutic target (mean difference = −1.52 days) aligns with pharmacokinetic principles. Continuous infusion allows for rapid achievement of steady-state concentrations, typically within 24 h, without the need for multiple dosing intervals to reach equilibrium. This is particularly advantageous in critically ill patients, where delayed attainment of therapeutic concentrations may be associated with treatment failure and the emergence of resistance. The faster time to target observed in our analysis—more than 1 day shorter with CI—could have meaningful implications for patients with severe infections such as MRSA bacteremia or endocarditis, where early appropriate antimicrobial therapy is strongly associated with improved outcomes (Abdelmessih et al., 2022; Lucian et al., 2026). However, it is important to note that although target attainment is a validated surrogate marker, its relationship with clinical outcomes in the context of CI versus II remains incompletely characterized. The modest heterogeneity observed for target attainment (I^2^ = 38%) was partly attributable to differences in target definitions across studies, with some employing traditional trough-based targets for II and steady-state plateau concentrations for CI, while others adopted AUC/MIC-guided targets. The shift toward AUC-guided dosing, endorsed by the 2020 IDSA guidelines (Reyes Curcio et al., 2026; Yıldız et al., 2024), may reduce the distinction between CI and II in terms of target attainment as AUC can be effectively achieved with either infusion strategy when therapeutic drug monitoring is optimized.

Despite the clear pharmacokinetic and renal safety advantages of CI, our analysis found no significant differences in clinical efficacy, microbiological clearance, or mortality between the two infusion modalities. The pooled odds ratios for these outcomes were all close to unity, with narrow confidence intervals and minimal heterogeneity. This observation warrants careful interpretation (Garnier et al., 2006). Several explanations may account for this apparent disconnect. First, the studies included in this analysis enrolled patients with diverse types and severities of infections, ranging from relatively mild OPAT cases to critically ill ICU patients with septic shock (Musta et al., 2009). The overall effect on clinical outcomes may be diluted by this heterogeneity, particularly if the relationship between pharmacokinetic optimization and clinical response varies by infection type and host factors (Brown et al., 2012). Second, although CI improves target attainment, the absolute difference in exposure may be insufficient to alter clinical outcomes when vancomycin is used for infections where the MIC is low and standard II regimens already achieve adequate exposure (Hos et al., 2017). Third, the studies may have been underpowered to detect modest differences in clinical outcomes as most were designed primarily to assess pharmacokinetic endpoints or nephrotoxicity. The low event rates for mortality and microbiological failure in many studies further limit the ability to detect meaningful differences (Truong et al., 2018). Fourth, clinical efficacy and mortality are influenced by numerous factors beyond vancomycin dosing, including adequacy of source control, timing of therapy, severity of underlying illness, and the use of concomitant antimicrobial agents (Trinh et al., 2018). It is possible that any potential benefit of CI on infection-related outcomes is overshadowed by these confounders, particularly in observational studies where residual confounding may persist despite propensity score matching.

The subgroup analyses conducted in this review did not reveal significant differences in treatment effects across study designs, patient populations, or dosing strategies. Although the point estimates for nephrotoxicity were similar between RCTs and observational studies, the confidence intervals in the RCT subgroup were wider due to the limited number of randomized trials, precluding definitive conclusions. The absence of significant interaction by population (ICU versus non-ICU) suggests that the benefits of CI may extend across the spectrum of illness severity, although the number of studies available for stratified analyses was modest. Similarly, the comparison of AUC-guided versus trough-guided dosing strategies did not demonstrate significant differences in outcomes, but this analysis was limited by the small number of studies that explicitly adopted AUC-guided monitoring (Lou et al., 2024a). As AUC-guided dosing becomes more widely implemented, future studies may clarify whether the advantages of CI persist or diminish when both arms are managed with optimized therapeutic drug monitoring.

The findings of this meta-analysis must be interpreted in the context of several limitations. First, the majority of included studies were observational in design, with only four RCTs identified. Although we assessed risk of bias using appropriate tools and found most studies to have moderate or low risk, the potential for confounding by indication remains a concern. Patients receiving CI may differ systematically from those receiving II in ways that influence outcomes, such as illness severity, renal function at baseline, or institutional protocols. Although several studies used propensity score matching to mitigate this bias, residual confounding cannot be entirely excluded (Puga et al., 2026). Second, substantial heterogeneity was observed for some outcomes, particularly time to therapeutic target (I^2^ = 67%), and the sources of this heterogeneity were not fully explained by prespecified subgroup analyses. Variability in target concentration definitions, frequency of therapeutic drug monitoring, and institutional practices likely contributed to this heterogeneity (Alsuwaylihi et al., 2026). Third, outcome definitions varied across studies, particularly for nephrotoxicity and clinical efficacy. Although we harmonized definitions as much as possible, differences in thresholds and timing of assessment may have introduced variability (Lou et al., 2024b). Fourth, publication bias was detected for target attainment, with funnel plot asymmetry and a significant Egger’s test. However, trim-and-fill analysis did not alter the direction or significance of the effect, suggesting that although publication bias may exist, it does not invalidate the finding. Fifth, the generalizability of our findings to certain populations—such as neonates, patients receiving renal replacement therapy, or those with severe burns—may be limited by the small number of studies in these subgroups (Lou et al., 2024c). Finally, the relatively short follow-up durations in most studies (typically hospital discharge or end of therapy) mean that longer-term outcomes, including persistent renal impairment or late recurrence of infection, were not captured. Heterogeneity in dosing regimens and TDM-guided dose adjustment strategies across studies represents an important limitation. Although we extracted detailed dosing information, the variability in institutional protocols for dose adjustment may have influenced outcomes.

Despite these limitations, this meta-analysis has several strengths. It represents the most comprehensive synthesis so far, incorporating studies published through March 2026 and including a large total sample of more than 5,000 patients. The rigorous methodology, including systematic risk-of-bias assessment, prespecified subgroup analyses, and comprehensive publication bias evaluation, enhances the reliability of the findings. The inclusion of both RCTs and observational studies allows for assessment of consistency across study designs, and the robust sensitivity analyses confirm the stability of the primary results. Furthermore, the focus on contemporary outcomes, including target attainment defined by both trough-based and AUC-guided criteria, reflects the evolving landscape of vancomycin therapeutic monitoring.

The findings of this meta-analysis have important clinical implications. For patients at high risk of nephrotoxicity—including those with pre-existing renal impairment, those receiving concomitant nephrotoxic agents such as aminoglycosides or amphotericin (McCafferty et al., 2026), and critically ill patients with hemodynamic instability—CI may offer a safer alternative to II without compromising efficacy (Huang et al., 2026). The faster achievement of therapeutic targets with CI may be particularly valuable in the initial management of severe infections, where delays in effective therapy are associated with increased mortality. However, the implementation of CI requires careful consideration of practical factors. CI necessitates the use of a dedicated intravenous line and a continuous infusion pump, which may not be feasible in all settings, particularly in outpatient or resource-limited environments (Jeyaraj et al., 2026). Additionally, CI requires frequent monitoring to ensure stable concentrations, and interruptions in infusion (e.g., for surgical procedures or line changes) can lead to subtherapeutic periods that may not occur with II. The choice between CI and II should, therefore, be individualized, taking into account patient characteristics, institutional resources, and the specific clinical context.

Future research should prioritize large, well-designed randomized controlled trials with standardized outcome definitions, particularly for clinical efficacy and mortality. Such trials should incorporate modern AUC-guided therapeutic drug monitoring in both arms to determine whether the benefits of CI persist when optimized monitoring is applied uniformly. Additionally, studies focusing on specific high-risk populations—such as patients with baseline renal impairment, those receiving prolonged vancomycin therapy, or those with deep-seated infections such as endocarditis or osteomyelitis—would help clarify which subgroups derive the greatest benefit from CI. Cost-effectiveness analyses are also needed to inform resource allocation as the potential reduction in nephrotoxicity with CI must be weighed against the increased logistical demands and monitoring costs.

## Conclusion

This meta-analysis demonstrates that continuous infusion of vancomycin significantly reduces nephrotoxicity, improves target concentration attainment, and shortens time to therapeutic target compared with intermittent infusion, while clinical efficacy and mortality remain comparable. These findings support CI as a preferred strategy in patients at elevated risk of renal injury, particularly in intensive care and burn settings. Further randomized controlled trials with standardized outcome definitions and AUC-guided monitoring are warranted to confirm these benefits.

## Data Availability

The original contributions presented in the study are included in the article/Supplementary Material; further inquiries can be directed to the corresponding author.
